# 25-Hydroxycholesterol-Conjugated EK1 Peptide with Potent and Broad-Spectrum Inhibitory Activity against SARS-CoV-2, Its Variants of Concern, and Other Human Coronaviruses

**DOI:** 10.3390/ijms222111869

**Published:** 2021-11-01

**Authors:** Qiaoshuai Lan, Chao Wang, Jie Zhou, Lijue Wang, Fanke Jiao, Yanbo Zhang, Yanxing Cai, Lu Lu, Shuai Xia, Shibo Jiang

**Affiliations:** 1 Key Laboratory of Medical Molecular Virology (MOE/NHC/CAMS), School of Basic Medical Sciences, Shanghai Institute of Infectious Disease and Biosecurity, Fudan University, 130 Dong An Road, Shanghai 200032, China; 18111010010@fudan.edu.cn (Q.L.); 19211010046@fudan.edu.cn (J.Z.); 19111010061@fudan.edu.cn (L.W.); 20111010053@fudan.edu.cn (F.J.); 19301050218@fudan.edu.cn (Y.Z.); 18111010051@fudan.edu.cn (Y.C.); lul@fudan.edu.cn (L.L.); 2 State Key Laboratory of Toxicology and Medical Countermeasures, Beijing Institute of Pharmacology and Toxicology, 27 Tai-Ping Road, Beijing 100850, China; chaow301@sina.com

**Keywords:** human coronavirus, 25-Hydroxycholesterol, fusion inhibitor, lipopeptide

## Abstract

The COVID-19 pandemic caused by SARS-CoV-2 infection poses a serious threat to global public health and the economy. The enzymatic product of cholesterol 25-hydroxylase (CH25H), 25-Hydroxycholesterol (25-HC), was reported to have potent anti-SARS-CoV-2 activity. Here, we found that the combination of 25-HC with EK1 peptide, a pan-coronavirus (CoV) fusion inhibitor, showed a synergistic antiviral activity. We then used the method of 25-HC modification to design and synthesize a series of 25-HC-modified peptides and found that a 25-HC-modified EK1 peptide (EK1P4HC) was highly effective against infections caused by SARS-CoV-2, its variants of concern (VOCs), and other human CoVs, such as HCoV-OC43 and HCoV-229E. EK1P4HC could protect newborn mice from lethal HCoV-OC43 infection, suggesting that conjugation of 25-HC with a peptide-based viral inhibitor was a feasible and universal strategy to improve its antiviral activity.

## 1. Introduction

As of now, coronavirus disease 2019 (COVID-19), caused by the severe acute respiratory syndrome coronavirus 2 (SARS-CoV-2), has resulted in more than 238 million confirmed cases and 4.86 million deaths worldwide (https://COVID19.who.int, accessed on 15 September 2021). Although the application of COVID-19 vaccines has shown significant efficacy in the reduction in hospitalization and deaths, pan-HCoV inhibitor-based prophylactics and therapeutics remain inadequate [1].

Similar to SARS-CoV-2, SARS-CoV and Middle East respiratory syndrome coronavirus (MERS-CoV) are also recognized as highly pathogenic human coronaviruses (HCoVs) [2,3]. It is worth noting that new cases of MERS-CoV infection are continuously reported in the Middle East [4]. Low-pathogenic HCoVs, including HCoV-OC43, HCoV-229E, HCoV-NL63, and HCoV-HKU1, are distributed worldwide and can cause mild infectious diseases in humans [5]. Therefore, it is essential to develop effective pan-CoV inhibitors against HCoVs [6]. 

Viral entry is an important process of the viral life cycle. The HCoV entry process consists of several steps: (1) receptor binding, (2) proteolytic activation of the spike (S) protein, and (3) virus fusion with and entry into the host cell. All of these steps can serve as targets for the development of viral fusion/entry inhibitors [7].

A key step of the membrane fusion process is the formation of the six-helix bundle (6-HB) between the heptad repeat 1 (HR1) and HR2 domains in the S protein S2 subunit [8]. Because the sequence and interaction models of the HR1 and HR2 domains are highly conserved in different HCoVs, the 6-HB formation is considered an important target for the development of pan-CoV fusion/entry inhibitors. Our previous study demonstrated an EK1 peptide to be a pan-CoV fusion inhibitor able to bind with the HR1 domains of HCoVs such as SARS-CoV, MERS-CoV, HCoV-OC43, HCoV-229E, and HCoV-NL63. Such binding results in the formation of heterologous 6-HB competitively block the homologous 6-HB formation between the HR1 and HR2 of the corresponding HCoVs. This, in turn, blocks the fusion with and entry into the host cell [9].

The enzymatic product of interferon-stimulated gene cholesterol 25-hydroxylase (CH25H), 25-Hydroxycholesterol (25-HC) plays an important role in sterol metabolism, as well as innate and adaptive immunity [10]; 25-HC can inhibit the infection of various human and animal viruses, including vesicular stomatitis virus (VSV), Ebola virus (EBOV), and HIV-1 [10,11]. Recently, 25-HC showed the inhibitory activity against infections by SARS-CoV-2 and other HCoVs by blocking viral membrane fusion through the suppression of cholesterol export, thereby reducing the membrane cholesterol level [12,13]. Mechanistic studies showed that 25-HC could indeed inhibit viral membrane fusion [12,13].

In the present study, we found that the combination of 25-HC with an EK1 peptide showed a synergistic effect against the infections by SARS-CoV-2 and MERS-CoV pseudovirus (PsV). We then used different flexible linkers to conjugate 25-HC to EK1 and generated four lipopeptides: EK1P4HC, EK1P8HC, EK1P12HC, and EK1P24HC, each of which exhibited a potent anti-SARS-CoV-2 activity. Among these four lipopeptides, EK1P4HC showed the most potent inhibitory activity against the infection by pseudotyped SARS-CoV-2, SARS-CoV, and MERS-CoV, as well as authentic HCoV-OC43 and HCoV-229E. Importantly, EK1P4HC could also potently inhibit the infection of four SARS-CoV-2 variants of concern (VOCs) and protect newborn mice from lethal HCoV-OC43 infection. These results suggest that the 25-HC modification for peptide-based viral fusion/entry inhibitors is a feasible and universal approach to design bifunctional inhibitors with improved antiviral activity.

## 2. Results

### 2.1. The Combination of 25-HC and EK1 Peptide Showed the Synergistic Antiviral Effect

We first measured the HCoV inhibitory activity of 25-HC in our established pseudovirus (PsV) and authentic virus infection systems. We found that 25-HC could potently inhibit authentic HCoV-229E infection in the Huh-7 cell line with a half-maximal inhibitory concentration (IC50) value of 5.35 μM (Supplementary Figure S1). Similarly, 25-HC could effectively inhibit SARS-CoV-2, SARS-CoV, and MERS-CoV PsV infections (Supplementary Figure S1) with IC50 values of 4.11, 2.79, and 3.95 μM, respectively. 

**Figure 1:**
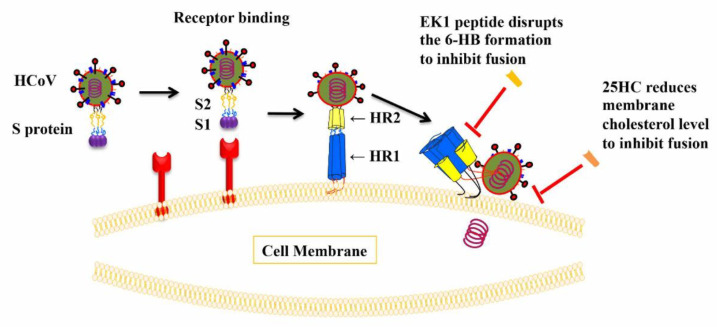
Entry process of HCoVs and inhibitory targets for EK1 and 25-HC. The entry process of HCoVs includes receptor binding and membrane fusion. Coronavirus spike protein is the main viral protein responsible for the entry of HCoVs and it serves as a valuable target for the development of antivirals. The receptor binding process is mainly mediated by the viral receptor-binding domain (RBD) which is located in the S1 subunit of the spike protein, while the key step of membrane fusion is mediated by the formation of 6-HB structure (interaction of HR1 and HR2 domains). EK1 peptide targeting the HR1 domain can disrupt viral 6-HB formation to inhibit membrane fusion, and 25-HC reduces membrane cholesterol to inhibit membrane fusion, thus indicating distinctly different mechanisms of action.

**Figure 2:**
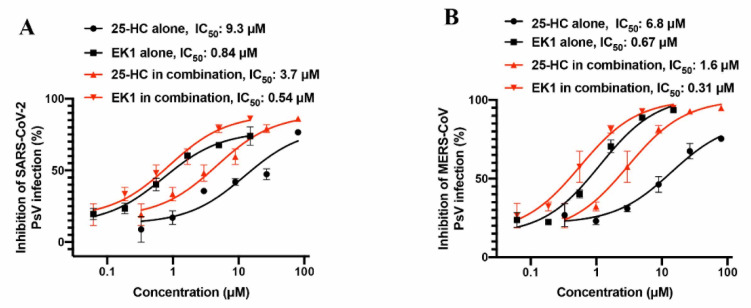
Combinations of 25-HC and EK1 show improved HCoV inhibitory activity. (**A**) Combinations of 25-HC and EK1 show improved HCoV inhibitory activity against SARS-CoV-2 PsV. (**B**) Combinations of 25-HC and EK1 show improved HCoV inhibitory activity against MERS-CoV PsV. The effective concentrations of EK1 or 25-HC tested alone or in combination for inhibiting SARS-CoV-2 or MERS-CoV pseudovirus infection are plotted with two curves. The black curves represent the inhibitory activity of EK1 or 25-HC tested alone, while the red curves represent the inhibitory activity of EK1 or 25-HC tested in EK1/25-HC combination. Each sample was tested in duplicate and experiments were repeated twice.

Based on previous studies, 25-HC could inhibit HCoV entry by reducing the cholesterol level in the membranes of target cells, while EK1 peptide could inhibit HCoV infection by blocking the viral 6-HB formation (Figure 1). Since these two molecules inhibited virus entry through different mechanisms of action, we asked if combining 25-HC and EK1 might have a synergistic effect. Thus, we assessed the inhibitory activity of 25-HC/EK1 in combination, 25-HC alone, and EK1 alone against SARS-CoV-2 PsV infection. We found that the combinations of 25-HC and EK1 showed a significant synergy with a combination index (CI) of 0.8 and a dose reduction of 3- and 2-fold, respectively (Figure 2A). Similarly, the combination of 25-HC and EK1 also exhibited a synergistic effect with a CI of 0.7 and a dose reduction of 4- and 2-fold, respectively, against MERS-CoV PsV infection (Figure 2B). These results confirm that 25-HC and EK1 could inhibit SARS-CoV-2 and MERS-CoV infection through different mechanisms of action.

### 2.2. Construction and Evaluation of 25-HC Conjugated EK1-Based Lipopeptides with Different Linkers

**Figure 3:**
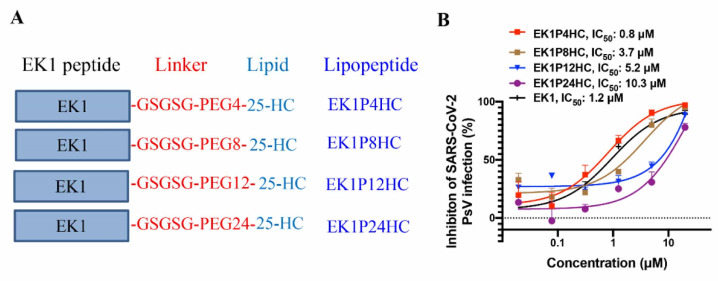
Sequence and SARS-CoV-2 PsV inhibitory activity of 25-HC-modified peptides. (**A**) The sequence of 25-HC-modified peptides. (**B**) SARS-CoV-2 PsV inhibitory activity of 25-HC-modified peptides. Each peptide was tested in triplicate, and experiments were repeated twice.

As demonstrated in Section 2.1 above, 25-HC combined with EK1 exhibits a synergistic antiviral effect, and we have already shown the much-improved anti-HCoV activity of EK1-based lipopeptide (EK1C4) [9]. Therefore, we designed and constructed a series of 25-HC-modified, EK1-based lipopeptides, including EK1P4HC, EK1P8HC, EK1P12HC, and EK1P24HC, in which 25-HC was conjugated to the C terminus of the EK1 peptide with different flexible linkers (Figure 3A). We found that EK1P4HC showed the most potent SARS-CoV-2 PsV inhibitory activity with an IC50 value of 0.8 μM (Figure 3B); nonetheless, EK1P8HC, EK1P12HC, EK1P24HC, and EK1 were also effective in inhibiting SARS-CoV-2 PsV infection with IC50 values of 3.7, 5.2, 10.3, and 1.2 μM, respectively. Meanwhile, we measured the cytotoxicity of those lipopeptides in the target cells and found no apparent cytotoxicity at concentrations of up to 20 μM (Supplementary Figure S2). We then chose EK1P4HC for the subsequent studies.

### 2.3. EK1P4HC Potently Inhibited Infection by SARS-CoV-2 and Its Variants

**Figure 4:**
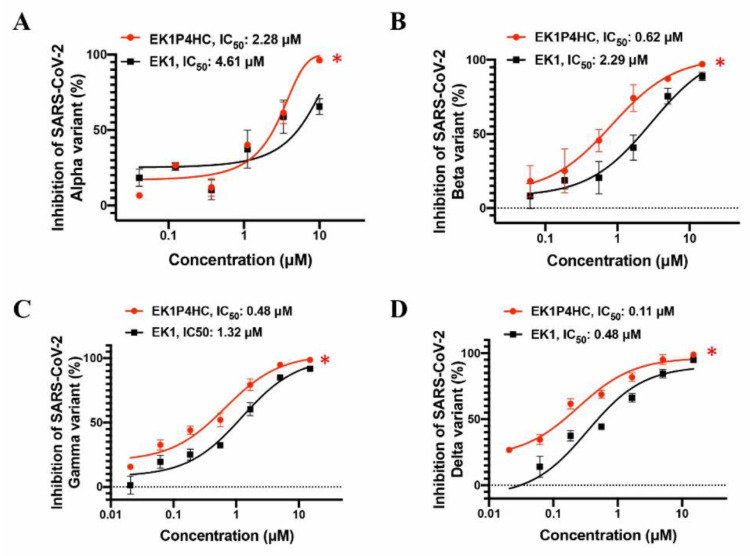
EK1P4HC could potently inhibit the infections by SARS-CoV-2 VOCs. Inhibitory activity of EK1P4HC against SARS-CoV-2 VOCs, including Alpha (**A**), Beta (**B**), Gamma (**C**), and Delta (**D**) variants. Each peptide was tested in triplicate, and experiments were repeated twice. Statistical differences were analyzed at concentrations of 1.66 μM or 10 μM of peptides, *, *p* < 0.05.

Next, we evaluated the inhibitory activity of EK1P4HC against infection by SARS-CoV-2 VOCs, namely B.1.1.7 (Alpha), B.1.351 (Beta), P.1 (Gamma), and B.1.617.2 (Delta). We found that EK1P4HC could also potently inhibit the infection of these SARS-CoV-2 VOC PsVs with IC50 values ranging from 0.11 to 2.28 μM, while EK1 peptide alone inhibited their infection with IC50 values ranging between 0.48 and 4.61 μM (Figure 4A–D). These results suggest that EK1P4HC is a potent lipopeptide capable of effectively inhibiting the infections by SARS-CoV-2 and its VOCs.

### 2.4. EK1P4HC Exhibited a Potent Inhibitory Activity against Infection by Authentic HCoV-229E and HCoV-OC43, as Well as Pseudotyped SARS-CoV and MERS-CoV

**Figure 5:**
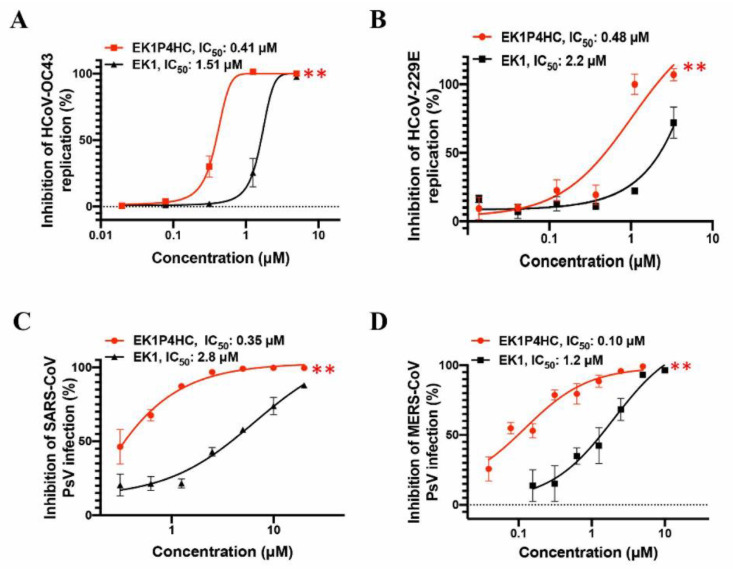
Broad HCoV inhibitory activity of EK1P4HC. (**A**) Inhibitory activity of EK1P4HC against HCoV-OC43 replication. (**B**) Inhibitory activity of EK1P4HC against HCoV-229E replication. (**C**) Inhibitory activity of EK1P4HC against SARS-CoV PsV infection. (**D**) Inhibitory activity of EK1P4HC against MERS-CoV PsV infection. Each peptide was tested in triplicate, and experiments were repeated twice. Statistical differences were analyzed at the concentration of 1.25 μM of peptides, **, *p* < 0.01.

We then assessed the inhibitory activity of EK1P4HC against the infection of other HCoVs, including the authentic HCoV-229E and HCoV-OC43 and pseudotyped SARS-CoV and MERS-CoV. Compared with the original peptide EK1, EK1P4HC showed an improved inhibitory activity against authentic HCoV-OC43 and HCoV-229E infection with IC50 values of 0.41 and 0.48 μM, respectively (Figure 5A,B). Similarly, EK1P4HC was much more effective than EK1 against SARS-CoV and MERS-CoV PsV infection with IC50 values of 0.35 and 0.10 μM, respectively (Figure 5C,D).

### 2.5. EK1P4HC Protected Newborn Mice from Lethal HCoV-OC43 Infection

**Figure 6:**
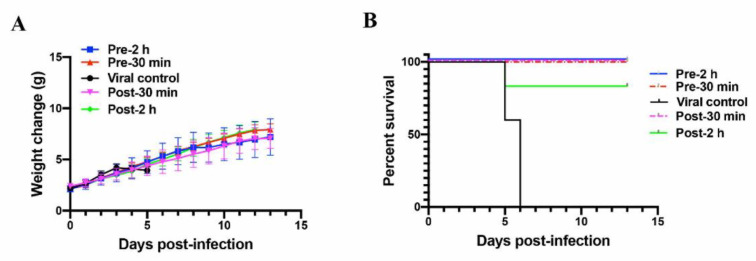
In vivo HCoV-OC43 inhibitory activity of EK1P4HC. (**A**) Bodyweight change of HCoV-OC43-infected newborn mice. (**B**) Survival curves of HCoV-OC43-infected newborn mice.

To further assess the in vivo protective efficacy of EK1P4HC, we performed HCoV-OC43 challenge experiments using a newborn mouse model [14]. We first assessed the protective efficacy of high-dose EK1P4HC (10 mg/kg) [9]. EK1P4HC was given by intranasal administration at 0.5 and 2 h before (prophylactic-0.5 group and prophylactic-2 group) or after (therapeutic-0.5 group and therapeutic-2 group) the challenge of HCoV-OC43 (100 TCID50). The bodyweight change and survival rates of these treated newborn mice were observed until the 14th day post-infection. As shown in Figure 6A, the bodyweight of the mice in the viral control group began to decrease from the 5th day post-infection, and all mice died on the 6th day post-infection. At the same time, the bodyweight of the newborn mice in the prophylactic and therapeutic groups steadily increased. The newborn mice in the prophylactic-0.5, prophylactic-2, and therapeutic-0.5 groups were 100% protected from lethal HCoV-OC43 infection, while the survival rate of the newborn mice in the therapeutic-2 group was 83% (Figure 6B). These results suggested that EK1P4HC had excellent in vivo protective efficacy.

We further assessed the protective efficacy of low-dose EK1P4HC (1 mg/kg) against HCoV-OC43 infection. EK1P4HC was also intranasally administered at 0.5 and 2 h before (prophylactic-0.5 group and prophylactic-2 group) or after (therapeutic-0.5 group and therapeutic-2 group) the challenge of HCoV-OC43, and both bodyweight and survival rate were observed. Compared with the viral control group, the average bodyweights of the newborn mice in the prophylactic-0.5 group and prophylactic-2 group showed no significant decrease, and the survival rates of the newborn mice in the prophylactic-0.5 group and prophylactic-2 group were 71% and 60%, respectively (Supplementary Figure S3). The low-dose EK1P4HC administration did not increase mouse survival rate in the therapeutic groups, but it did increase survival time, suggesting that low-dose EK1P4HC still has a certain protective effect (Supplementary Figure S3).

## 3. Discussion

After the outbreak of COVID-19, many nonspecific antiviral drugs and repurposed drugs were used in hospitals for the treatment of COVID-19 patients [15,16]. However, their clinical application remains controversial due to the disparity of their therapeutic efficacy and side effects. Neutralizing antibodies with potent SARS-CoV-2 inhibitory activity were urgently developed, and while some patients receiving these treatments showed an improvement in clinical outcomes and a decrease in viral load, others did not [17,18]. As an additional complication, the emergence of SARS-CoV-2 VOCs posed still another challenge to the application of these neutralizing antibodies [19], essentially because many of them could compromise the neutralizing antibodies [20]. Interestingly, EK1 and EK1C4 peptides showed potent inhibitory activity against several SARS-CoV-2 VOCs tested [19,21].

Given that the EK1 peptide inhibits HCoV fusion by blocking viral 6-HB formation, while 25-HC suppresses HCoV fusion by reducing the membrane cholesterol level, we proposed that combining 25-HC with EK1 might result in an antiviral synergy. As expected, 25-HC/EK1 in combination did show a synergistic antiviral activity against SARS-CoV-2 and MERS-CoV PsV infections. 

Consequently, we were encouraged to design and construct several lipopeptides by conjugating 25-HC to the C terminus of the EK1 peptide using different linkers to augment the anti-HCoV activity of the EK1 peptide based on two suppositions: (1) that 25-HC/EK1 in combination would have a synergistic antiviral effect and (2) that the EK1-based lipopeptide, e.g., EK1C4, would improve anti-HCoV activity over its competing EK1 lipopeptide candidates. Indeed, one such 25-HC-modified, EK1-based lipopeptide, EK1P4HC, exhibited a more potent inhibitory activity than EK1 against infection by SARS-CoV-2 and its 4 VOCs, as well as other HCoVs, including SARS-CoV, MERS-CoV, HCoV-229E, and HCoV-OC43. Notably, the inhibitory activity of EK1P4HC against SARS-CoV-2 infection was significantly lower than those of the cholesterol-conjugated EK1 (e.g., EK1C4) and the palmitic acid-conjugated EK1 (e.g., EK1P) [9], possibly because 25-HC in an EK1P4HC was less effective than cholesterol in EK1C4, and palmitic acid in EK1P, in binding to the lipid membranes to mediate the inhibition of the virus–cell fusion. However, EK1P4HC at 10 mg/kg exhibited a similarly high in vivo efficacy as EK1C4 in protecting newborn mice against a lethal HCoV-OC43 infection through intranasal administration, indicating that EK1P4HC had a similar potential as EK1C4 to be further developed as a new antiviral agent for preventing and treating the infections by SARS-CoV-2 and its VOCs, as well as other highly pathogenic HCoVs. It can also be used to treat some aged or immunodeficient patients with potentially lethal, low-pathogenic HCoVs, such as HCoV-229E and HCoV-OC43. This study also provides a feasible and universal strategy to improve the antiviral activity of peptide-based fusion/entry inhibitors against other highly pathogenic enveloped viruses, such as HIV-1, Nipah virus, and Ebola virus.

The main advantage of EK1P4HC over 25-HC, EK1, or EK1C4 used alone is that EK1P4HC targets two different sites when mediating virus–cell fusion, thus having a higher genetic barrier to resistance than 25-HC, EK1, or EK1C4, which targets only one site for inhibiting virus–cell fusion. EK1P4HC is expected to be effective against the SARS-CoV-2 mutants resistant to 25-HC, EK1, or EK1C4, if such mutants emerge in the future. 

However, some limitations to our study of 25-HC-modified peptides did arise. For example, the solubility of 25-HC-modified, EK1-based lipopeptide in PBS is lower than that of unmodified EK1 peptide. Some optimal solvents, used to increase the solubility of EK1P4HC in a physiological solution, must be identified before preclinical and clinical studies can begin. Our previous study showed that a suitable linker in cholesteryl-EK1 could benefit its antiviral potency. Here, we did notice that some linkers, such as GSGSG-PEG 8, 12, and 24, even weaken the inhibitory efficacy of 25-HC-conjugated EK1, suggesting that a greater number of different linkers should be tested in order to optimize the structure of the 25-HC-modified lipopeptides with improved antiviral activity.

Overall, in this study, we developed a novel strategy whereby 25-HC is used to modify peptide-based viral fusion/entry inhibitors to generate lipopeptides with improved antiviral activity by combinatorial effect. The 25-HC-modified EK1 peptide, EK1P4HC, with a potent and broad HCoV inhibitory activity in vitro and in vivo, shows promise for the development as a new pan-CoV fusion/entry inhibitor for the prevention and treatment of the infections by SARS-CoV-2 and, critically, its VOCs, and other highly pathogenic HCoVs.

## 4. Materials and Methods

### 4.1. Cells, Viruses, Peptides, and Plasmids

Cells used in this study, including 293T, Huh-7, RD, and Caco2 cell lines, were all cultured in Dulbecco’s Modified Eagle’s Medium (DMEM) containing 5% fetal bovine serum (FBS). HCoV-229E (VR-740) and HCoV-OC43 (VR-1558) virus strains were obtained from American Type Culture Collection (ATCC) and 25-Hydroxycholesterol (25-HC) was purchased from targetMOI. The 25-HC-modified peptides were provided by Dr. Chao Wang [22] (Table 1). Plasmids pNL4-3.Luc.R-E, pcDNA3.1-SARS-CoV-2-S, pcDNA3.1-SARS-CoV-S, and pcDNA3.1-MERS-CoV-S were kept in our laboratory.

### 4.2. Package of Coronavirus Pseudoviruses

Coronavirus pseudoviruses (PsVs) were packaged as previously reported [9]. HEK293T cells were co-transfected with two plasmids: HIV backbone plasmid (pNL4-3.Luc.R-E) and plasmid-expressing coronavirus spike protein (pcDNA3.1-SARS-CoV-2-S, pcDNA3.1-SARS-CoV-S, or pcDNA3.1-MERS-CoV-S). Fresh DMEM containing 5% FBS was supplemented at 12 h post-transfection. The supernatants containing PsV particles were collected at 60 h post-transfection and refrigerated at −80 ℃.

### 4.3. Authentic Virus Inhibition Assay

Inhibition of authentic coronaviruses was performed as previously reported [9]. Briefly, authentic HCoV-OC43 or HCoV-229E was coincubated with a test peptide at different concentrations at 37 °C for 30 min. Then, these authentic virus–peptide mixtures were added into target cells in a 96-well plate (Huh-7 cell line for HCoV-229E, RD cell line for HCoV-OC43). CCK8 solution was used to assess virus-induced cytopathy when the cytopathic effect (CPE) of DMSO-treated target cells was clear and the IC50 value was calculated.

### 4.4. PsV Inhibition Assay

Inhibitory activity of peptides against infection by SARS-CoV-2 and its VOCs, including B.1.1.7 (Alpha), B.1.351 (Beta), P.1 (Gamma), and B.1.617.2 (Delta), SARS-CoV, and MERS-CoV PsVs, was assessed in Caco2 cells. Briefly, PsV was coincubated with a test peptide at different concentrations at 37 °C for 30 min, and the PsV–peptide mixture was added to Caco2 cells in wells of a 96-well plate. About 12 h later, the culture supernatants were replaced with fresh DMEM containing 5% FBS, followed by coculture for 36 h. Luciferase activity was then measured according to instructions of the Luciferase Assay System (Promega, Madison, WI, USA), and the IC50 value was calculated.

### 4.5. Measurement of 25-HC Inhibitory Activity against HCoVs

Inhibitory activity of 25-HC against HCoVs was measured as previously reported [13], but with some modifications. Briefly, target cells in a 96-well plate were pretreated with 25-HC at different concentrations for 8–12 h, and then PsVs or authentic viruses were added. After 12 h incubation, the old medium containing 25-HC and virus particles was replaced with fresh DMEM containing 5% FBS. Luciferase activity was measured after 36 h for measurement of its inhibitory activity against HCoV PsVs, and CCK-8 solution was used for the analysis of its inhibitory activity against authentic HCoVs, followed by calculating the IC50 value.

### 4.6. Measurement of Combinatorial Inhibitory Activity of 25-HC and EK1 against HCoVs

Target cells in a 96-well plate were first pretreated with 25-HC at different concentrations for 8–12 h, and then PsVs, or mixtures containing EK1 and PsVs, were added into target cells. Forty-eight hours later, luciferase activity was measured, and the IC50 value was calculated. The combination index (CI) was calculated using CalcuSyn software kindly provided by Dr. T. C. Chou. CI > and <1 represent the antagonistic and synergistic effect, respectively [23].

### 4.7. Cytotoxicity Assay

Cytotoxicity of peptides was assessed by CCK-8 assay. Briefly, target cells in a 96-well plate were coincubated with the test peptides at the indicated concentration for 36 h. Then, the CCK-8 solution (10 μL/well) was added and absorbance was measured after 2 h.

### 4.8. Assessment of In Vivo Protective Efficacy of EK1P4HC against HCoV-OC43 in Newborn Mice

Measurements of protective efficacy of EK1P4HC against HCoV-OC43 in newborn mice were performed as previously reported [9]. Briefly, pregnant Balb/c mice were purchased from Shanghai Laboratory Animal Research Center, and related experiments were performed when the weight of the newborn mice reached 2 g. EK1P4HC in PBS was given by intranasal administration (10 mg/kg or 1 mg/kg) 0.5 and 2 h before or after the HCoV-OC43 challenge (100 TCID50). Bodyweight change and survival status of mice were observed until the 14th day post-infection.

### 4.9. Statistical Analysis

Survival curves of HCoV-OC43-infected mice and IC50 values in virus inhibition assays were produced by GraphPad Prism 8.0. Differences between groups were analyzed by using an unpaired t-test. *p* values < 0.05 were considered significant; *, *p* < 0.05; **, *p* < 0.01; ***, *p* < 0.001.

## Figures and Tables

**Table 1:** Sequences of 25-HC-modified EK1 peptides.

Name	Sequence	MW
**EK1P4HC**	SLDQINVTFLDLEYEMKKLEEAIKKLEESYIDLKEL**GSGSG-PEG4**-25-HC	5453
**EK1P8HC**	SLDQINVTFLDLEYEMKKLEEAIKKLEESYIDLKEL**GSGSG-PEG8**-25-HC	5689
**EK1P12HC**	SLDQINVTFLDLEYEMKKLEEAIKKLEESYIDLKEL**GSGSG-PEG12**-25-HC	5865
**EK1P24HC**	SLDQINVTFLDLEYEMKKLEEAIKKLEESYIDLKEL**GSGSG-PEG24**-25-HC	6393

Note: The sequence of EK1 is SLDQINVTFLDLEYEMKKLEEAIKKLEESYIDLKEL; 25-HC is attached to the C terminus by different linkers.

## Data Availability

The raw data of this article are available on request from the corresponding author.

## Supplementary Materials

The following are available online at https://www.mdpi.com/article/10.3390/ijms222111869/s1.
