# Supplementary material for: 25-Hydroxycholesterol-Conjugated EK1 Peptide with Potent and Broad-Spectrum Inhibitory Activity against SARS-CoV-2, Its Variants of Concern, and Other Human Coronaviruses
Source: Int J Mol Sci. 2021 Nov 1;22(21):11869. doi: 10.3390/ijms222111869 (PMC8584349; doi:10.3390/ijms222111869)
Supplement: Supplementary file 1 [file ijms-22-11869-s001.zip › ijms-1437454-supplementary.pdf]

Supplementary Materials for

25-Hydroxycholesterol-Conjugated EK1 Peptide with Potent and Broad-Spectrum Inhibitory Activity against SARS-CoV-2, Its Variants of Concern and Other Human Coronaviruses

Qiaoshuai Lan [1,†], Chao Wang [2,†], Jie Zhou [1], Lijue Wang [1], Fanke Jiao [1], Yanbo Zhang [1], Yanxing Cai [1], Lu Lu [1], Shuai Xia [1,*], and Shibo Jiang [1,*]

1 Key Laboratory of Medical Molecular Virology (MOE/NHC/CAMS), School of Basic Medical Sciences, Shanghai Institute of Infectious Disease and Biosecurity, Fudan University, 130 Dong An Road, Shanghai 200032, China; 18111010010@fudan.edu.cn (Q.L.); 19211010046@fudan.edu.cn (J.Z.); 19111010061@fudan.edu.cn (L.W.); 20111010053@fudan.edu.cn (F.J.); 19301050218@fudan.edu.cn (Y.Z.); 18111010051@fudan.edu.cn (Y.C.); lul@fudan.edu.cn (L.L.)

2 State Key Laboratory of Toxicology and Medical Countermeasures, Beijing Institute of Pharmacology and Toxicology, 27 Tai-Ping Road, Beijing 100850, China; chaow301@sina.com

* Correspondence: sxia15@fudan.edu.cn (S.X.); shibojiang@fudan.edu.cn (S.J.)

† These authors contributed equally to this work.

Figure legend

Supplementary Figure S1. Broad coronavirus inhibitory activity of 25-HC.

Supplementary Figure S2. Cytotoxicity of 25-HC-modified peptides.

Supplementary Figure S3. In vivo protective efficacy of low dosage EK1P4HC (1mg/kg) against HCoV-OC43 challenge in the newborn mice model.

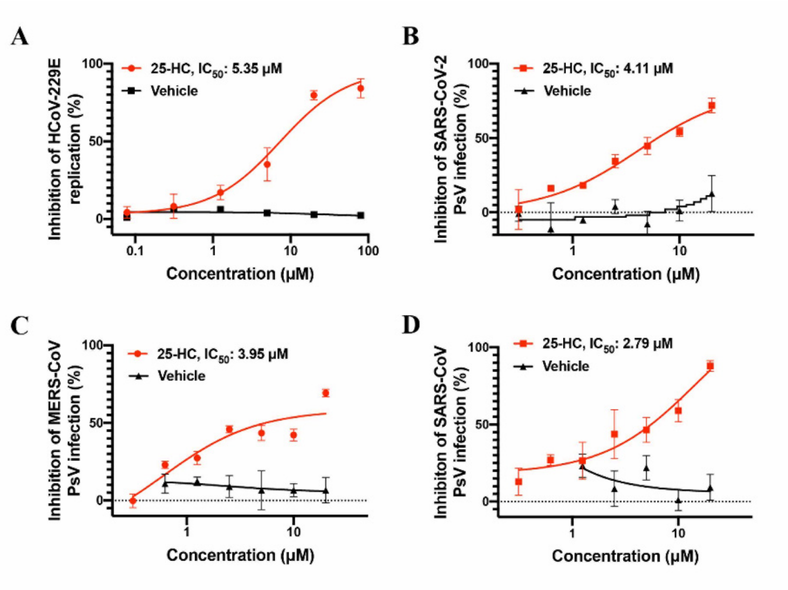

Supplementary Figure S1. Broad coronavirus inhibitory activity of 25-HC. (**A**) Inhibitory activity of 25-HC against authentic HCoV-229E infection. (**B**) Inhibitory activity of 25-HC against SARS-CoV-2 PsV infection. (**C**) Inhibitory activity of 25-HC against MERS-CoV PsV infection. (**D**) Inhibitory activity of 25-HC against SARS-CoV PsV infection.

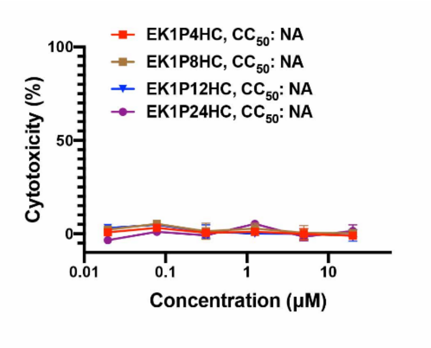

Supplementary Figure S2. Cytotoxicity of 25-HC-modified peptides.

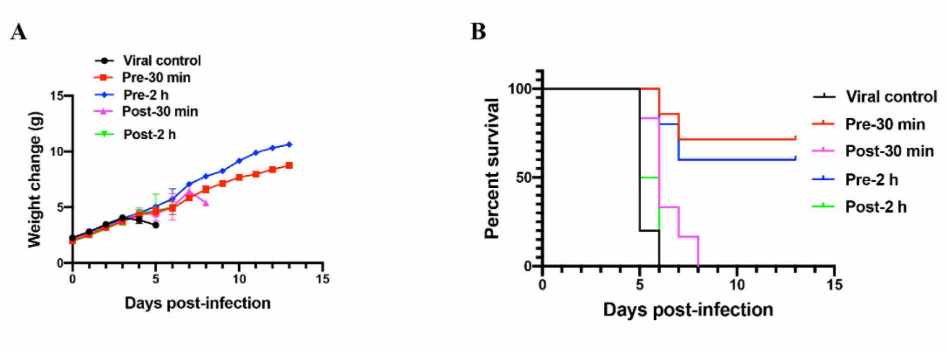

Supplementary Figure S3. In vivo protective efficacy of low dosage EK1P4HC (1mg/kg) against HCoV-OC43 challenge in the newborn mice model. (**A**) Body weight change of HCoV-OC43-infected newborn mice. (**B**) Survival curves of HCoV-OC43-infected newborn mice.
